# Early-Life Heat Stress Exposes Genotype-Dependent Male Fertility Limits in *Drosophila melanogaster* Under Sublethal Agrochemical Exposure

**DOI:** 10.3390/insects17040426

**Published:** 2026-04-16

**Authors:** David A. Sánchez-Rodríguez, Ying Ting Yang, Felipe Martelli, Nina Wedell

**Affiliations:** School of BioSciences, The University of Melbourne, Melbourne, VIC 3052, Australia; david.sanchezrodriguez@student.unimelb.edu.au (D.A.S.-R.);

**Keywords:** climate warming, insecticide contamination, imidacloprid, *Cyp6g1*, hormesis, early-life stress, fitness

## Abstract

Insect populations are increasingly exposed to concurrent climate warming and agrochemical contamination, yet how these stressors interact to influence reproductive performance remains poorly understood. Because fertility can constrain population growth before survival declines, understanding how environmental stress affects reproduction is essential for predicting demographic responses. Here, we investigated how elevated temperatures and sublethal imidacloprid exposure during development and early-life interact with the insecticide resistance locus *Cyp6g1* to influence male reproductive performance in *Drosophila melanogaster*. Males were reared from embryo to adulthood under factorial combinations of temperature and insecticide exposure, and mating behaviour and fertilisation success were subsequently quantified under benign assay conditions. Early-life heat reduced fertilisation success in a genotype-dependent manner, with a pronounced collapse observed in insecticide-susceptible males. Sublethal insecticide exposure modified this thermal response, restoring fertilisation success in susceptible males and producing non-additive interactions between thermal and agrochemical stress. In contrast, although mating frequency varied across treatments, it did not show the pronounced decline observed in fertilisation success, indicating that behavioural engagement does not necessarily predict functional reproductive output. These results suggest that environmental stress experienced during early-life can reshape reproductive performance, potentially through genotype-dependent shifts in physiological investment. Considering developmental stress history and genetic variation will therefore be important for predicting insect population responses to climate warming and environmental contamination.

## Simple Summary

Insect populations are declining worldwide, and environmental stressors such as rising temperatures and pesticide contamination are thought to contribute to these losses. However, these stressors rarely occur in isolation in nature, and their combined effects on reproduction are still poorly understood. In this study, we investigated how temperature and exposure to a low dose of a common insecticide affect male fertility in the fruit fly *Drosophila melanogaster*. Flies were raised from embryo to adulthood under different temperature and insecticide conditions and later tested for their ability to mate and produce offspring. We found that exposure to higher temperatures during development and early-life reduced reproductive success, particularly in males lacking a genetic variant that confers insecticide resistance. Surprisingly, low doses of insecticide sometimes altered this response. These results show that environmental stress during early life can have lasting effects on reproduction and that genetic differences among individuals can influence how insects respond to multiple environmental pressures. Understanding these interactions will help scientists better predict how insect populations may respond to climate change and chemical pollution.

## 1. Introduction

Insect populations are declining globally, with climate warming and agrochemical contamination recognised as major and interacting drivers of demographic instability [1]. Elevated temperatures disrupt insect physiology, alter life-history traits, and reduce reproductive performance [2,3], while agrochemical exposure imposes chronic energetic and toxicological stress [4,5]. Because warming and agrochemical exposure frequently co-occur in natural and agricultural landscapes, their combined effects are unlikely to be predictable from single-stressor studies [6,7]. Disentangling how these layered stressors interact to shape reproductive performance is therefore essential for understanding insect population resilience under climate change.

Reproduction represents a critical demographic bottleneck: reductions in fertility can constrain population growth even when survival remains unaffected [8]. Sublethal fertility impairment may therefore precede detectable declines in abundance, making reproductive performance a sensitive indicator of vulnerability [2,9]. Male reproductive function is particularly thermosensitive in insects, as elevated temperatures impair spermatogenesis, sperm viability, and fertilisation success, often below lethal thresholds [3,10,11]. Sublethal insecticide exposure can further influence reproductive traits by altering behaviour, metabolic allocation, oxidative balance, and stress-response pathways [9]. However, how thermal and agrochemical stress interact to shape male fertility under climate-relevant warming remains poorly resolved.

Pervasive insecticide use has driven the widespread evolution of resistance in both pest and non-pest insects, while overall pesticide toxicity in terrestrial systems continues to increase [12]. Resistance alleles frequently carry pleiotropic effects on fecundity, development, and life-history trade-offs, reflecting energetic costs or broader metabolic disruption [13,14,15,16]. Consequently, reproductive responses to environmental stress may vary across genotypes within a species. Yet whether resistance-associated genotypes differ in reproductive resilience to warming, and how agrochemical exposure modifies this relationship, remains largely unexplored.

In *Drosophila melanogaster*, variation at the *Cyp6g1* locus provides a well-characterised model of resistance evolution. The resistance-associated *Cyp6g1-BA* haplotype, defined by transposable element insertions and elevated gene expression, confers broad detoxification capacity relative to the ancestral susceptible *Cyp6g1-M* genotype [17,18]. Higher *Cyp6g1* expression levels confer resistance to several insecticide classes such as organochlorides, neonicotinoids, insect growth regulators, organophosphates, and carbamates [19,20,21,22]. Beyond detoxification, *Cyp6g1* variation has been linked to broader life-history effects, including fecundity, mating success, and developmental timing, suggesting potential pleiotropic consequences [23,24,25]. This system therefore offers a powerful framework to test whether resistance-associated genotypes differ in reproductive performance under interacting thermal and agrochemical stress.

Here, we investigated how exposure to elevated temperature and sublethal insecticide dose during development and early-life interacts with the *Cyp6g1* genotype to shape male reproductive performance in *Drosophila melanogaster*. Flies were reared from embryo to adulthood under factorial combinations of thermal and agrochemical stress, and mating rate and fertilisation success were quantified under non-stressful assay conditions to isolate early-life environmental history effects. We tested whether layered environmental stress reshapes reproductive limits and whether resistance genotype modifies vulnerability, expecting that the *Cyp6g1-M* genotype manifests increased vulnerability due to reduced xenobiotic capacity. Because reductions in fertilisation capacity directly constrain effective population size [2,26], this work links layered environmental stress, genetic variation, and demographic resilience under climate change.

## 2. Materials and Methods

### 2.1. Fly Resources

The Canton-S strain carrying the ancestral *Cyp6g1-M* allele was obtained from the Bloomington Drosophila Stock Center (BDSC #64349). An introgressed Canton-S line carrying the resistance-associated *Cyp6g1-BA* haplotype was generated using the Hikone-R strain (BDSC #4267) as the donor, as previously described [27]. Briefly, the *Cyp6g1-BA* allele was introgressed into the Canton-S background through eight generations of backcrossing with molecular verification of genotype at each generation. Homozygous *Cyp6g1-BA* individuals were subsequently intercrossed to establish the final introgressed population. Thus, this study compared two strains sharing a common Canton-S genetic background but differing at the *Cyp6g1* locus, carrying either the susceptible (*Cyp6g1-M*) or resistance-associated (*Cyp6g1-BA*) haplotype.

### 2.2. Fly Rearing Conditions

Fly stocks used for embryo collection were maintained at 25 °C under standard laboratory conditions. A total of eight experimental groups were generated using a fully factorial design combining the *Cyp6g1* genotype (*Cyp6g1-M* or *Cyp6g1-BA*), insecticide exposure (no insecticide or imidacloprid 0.1 ppm), and temperature regime (average 18 °C or average 28 °C). Experimental males were reared on sugar–yeast medium [28]. To mimic environmentally relevant daily temperature fluctuations, individuals were maintained from the embryo stage to adulthood in controlled incubators under one of two temperature regimes: a cool condition fluctuating between 14 °C and 22 °C (average 18 °C) or a warm condition fluctuating between 24 °C and 32 °C (average 28 °C), both at 45% relative humidity [29,30]. While a 25 °C constant is a standard benchmark for laboratory *Drosophila* maintenance, we intentionally employed fluctuating regimes to better reflect the natural thermal environment and to examine the transition from a more favourable field-realistic conditions to a more stressful one. Newly eclosed naïve males were collected and maintained under their respective experimental temperature and insecticide treatments until 3 days of age, at which point they were used in fertility assays. Virgin females used in mating assays were standardised, maintained at 25 °C on control medium, and were not exposed to experimental temperature or insecticide treatments.

### 2.3. Insecticide Dilution and Preparation of Dosed Fly Medium

Analytical-grade imidacloprid (Sigma-Aldrich^®^, St. Louis, MO, USA) was used in all experiments. Imidacloprid was initially dissolved in dimethyl sulfoxide (DMSO) to prepare a 10,000 parts per million (ppm) stock solution, which was stored at −20 °C until use. Prior to dosing the fly medium, an intermediate 100 ppm imidacloprid solution was prepared in DMSO. For insecticide-treated food, 1 mL of the 100 ppm intermediate solution was added to 1 L of lukewarm sugar–yeast fly medium, yielding a final imidacloprid concentration of 0.1 ppm, a sublethal concentration [31]. Control food received an equivalent volume of DMSO (1 mL/L) added to the fly medium without insecticide.

### 2.4. Male Fertility Assay

Male fertility was assessed using a repeated mating assay designed to quantify mating performance and non-competitive fertilisation success under sustained reproductive demand. Male flies were reared from the embryo stage under the assigned experimental conditions (genotype × temperature × insecticide exposure). Upon eclosion, males were collected within 8 h and isolated individually in fresh food vials corresponding to their treatment group. Naïve males were maintained under their respective experimental conditions until 3 days of age. At 3 days post eclosion, individual males (n = 10 per experimental group) were transferred to labelled mating vials. Each male was paired with three 3–5-day-old virgin Canton-S (*Cyp6g1-M*) females reared under standard laboratory conditions (25 °C, no insecticide). Mating behaviour was directly observed for a 4 h period, and all copulation events were recorded. If a male successfully mated with all three females before the end of the 4 h observation window, an additional three virgin females were introduced to maintain continuous mating opportunity and avoid female limitation of mating frequency. This protocol was applied consistently across all experimental treatments, and all successful mating events were recorded. Consequently, the 4 h observation window served as the standardized unit of reproductive opportunity for all individuals. At the conclusion of the 4 h mating period, all females that had mated were individually transferred to fresh vials containing standard medium (no insecticide) and maintained at 25 °C. Following each daily mating assay, males were transferred to fresh standard medium (no insecticide) and maintained overnight at room temperature to allow recovery. The mating assay was repeated daily for five consecutive days for each male. Mated females were individually transferred to fresh vials and allowed to oviposit for 48 h before being cleared; 10 days after transfer, vials were scored for the presence or absence of progeny as a binary measure of fertilisation success. For each male, two response variables were recorded: (i) daily number of mating events observed; and (ii) total number of successful fertilisation events, defined as matings that resulted in progeny production (Table S1).

### 2.5. Statistical Analysis

All statistical analyses were performed in R (version 4.4.2). Male reproductive performance was analysed using generalized linear mixed models (GLMMs) and generalized linear models (GLMs) appropriate for the distribution of each response variable. All models included the fully factorial fixed effects of temperature (average 18 °C vs. average 28 °C), insecticide exposure (no insecticide vs. imidacloprid 0.1 ppm), genotype (*Cyp6g1-M* vs. *Cyp6g1-BA*), and their interactions. Daily mating counts (day 1–5) were analysed using a Poisson GLMM with day included as a fixed effect to model temporal trends. To account for repeated measures within individuals, Male ID was included as a random effect. Model fit was confirmed using the Akaike Information Criterion (AIC) and Bayesian Information Criterion (BIC) (Table S3). Model significance for interaction terms and the temporal factor (Day) was assessed using likelihood ratio tests (χ^2^ tests). The χ^2^ statistic for the genotype main effect was derived from the Wald statistic (z^2^). Effect sizes are presented as incidence rate ratios (IRRs) representing multiplicative changes in daily mating frequency relative to the reference category (18 °C, no insecticide, *Cyp6g1-M*). Post hoc pairwise comparisons were performed using Tukey’s Honestly Significant Difference (HSD) tests to identify specific differences between treatment combinations (Table S2). Fertilisation success was analysed as the number of successful matings relative to the total number of matings per male using a binomial GLM with a logit link function. Because fertilisation success was defined at the level of the individual male (i.e., a single aggregated observation per male), the data do not constitute a repeated-measures structure, and no random effect was included. This approach explicitly accounts for variation in the total number of mating events among individuals by using the total number of matings as the binomial denominator (n), thereby weighing each observation according to the number of mating events contributing to the estimate of fertilisation success. The response variable was specified as a two-column integer matrix of “successes” (vials with progeny) and “failures” (vials without progeny) for each male. This approach explicitly accounts for variation in the total number of matings among individuals by weighting the contribution of each male to the model’s likelihood based on their total number of mating events. The full factorial model including the three-way interaction was fitted, and model terms were evaluated using likelihood ratio tests (χ^2^ tests). Model adequacy was validated by calculating the dispersion ratio (Pearson χ^2^/residual *df*); the resulting ratio (1.589) confirmed the model appropriately accounted for variance in mating outcomes (Table S3). Given the mechanistic structure of the experimental design and evidence for higher-order interaction effects, the full interaction model was retained for interpretation. Predicted probabilities and 95% confidence intervals were obtained using the *emmeans* package and back-transformed from the logit scale. Figures display raw individual-level data alongside model-derived estimated marginal means and their corresponding 95% confidence intervals.

## 3. Results

### 3.1. Environmental Stressors Interact with the Insecticide Resistance Haplotype to Reshape Mating Behaviour

**Figure 1:**
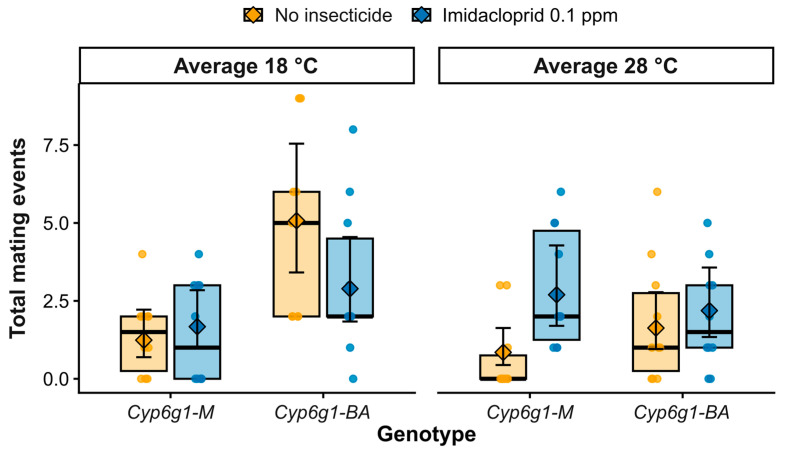
Genotype- and environment-dependent effects on male mating frequency. Total number of mating events per male accumulated across five consecutive daily 4 h mating assays under factorial combinations of temperature (average 18 °C or average 28 °C), insecticide exposure (no insecticide or imidacloprid 0.1 ppm), and Genotype (susceptible *Cyp6g1-M* haplotype or resistant *Cyp6g1-BA* haplotype). Points represent individual males (n = 10 males per group), with colours indicating treatment (orange = no insecticide; blue = imidacloprid 0.1 ppm). Boxes show the interquartile range of raw data (without whiskers). Diamonds indicate model-estimated marginal means, and error bars represent 95% confidence intervals derived from a negative binomial generalized linear model. Model predictions are back-transformed from the log scale. Full model results are provided in Table 1, with pairwise comparisons and model diagnostics available in Tables S2 and S3, respectively.

To assess how thermal and sublethal agrochemical stresses interact with genotype to shape male reproductive performance, flies were reared from embryo to adulthood under factorial combinations of temperature (average 18 °C or average 28 °C, mimicking environmentally relevant daily temperature fluctuations), insecticide exposure (no insecticide or 0.1 ppm imidacloprid), and genotype (*Cyp6g1-M* susceptible haplotype or *Cyp6g1-BA* resistance haplotype), generating eight treatment groups (Figure 1). Male reproductive output was quantified across five consecutive daily mating assays (Table S1). Daily mating frequency was primarily driven by a significant genotype effect (χ^2^_1_ = 16.46, *p* < 0.001; Table 1) and a strong temporal effect of day (χ^2^_4_ = 32.17, *p* < 0.001; Table 1). We observed a significant temperature × insecticide exposure interaction (χ^2^_1_ = 5.80, *p* < 0.016; Table 1), but no significant interaction between temperature and genotype or three-way interaction was observed (Table 1).

Under the cooler condition and no insecticide, *Cyp6g1-BA* males exhibited markedly elevated mating activity relative to *Cyp6g1-M* males (model-estimated means: 5.07 vs. 1.24 matings), corresponding to an approximately four-fold increase in mating frequency (Figure 1). Mating events reduced substantially under imidacloprid exposure in *Cyp6g1-BA* males, declining from 5.07 to 2.89 matings (approximately 43% reduction), whereas *Cyp6g1-M* males remained consistently low across exposure conditions.

Under the warmer condition, overall mating frequency declined and genotype differences were attenuated (Figure 1). In the absence of insecticide, mating activity in *Cyp6g1-BA* males decreased by approximately 68%, from 5.07 (cooler condition) to 1.63 matings (warmer condition), while *Cyp6g1-M* males showed comparatively modest changes across temperatures. Under combined heat and insecticide exposure, mating frequencies converged between genotypes (2.19 vs. 2.70 matings for resistance *Cyp6g1-BA* and susceptible *Cyp6g1-M* males, respectively).

The high significance of the day effect (*p* < 0.001) reflects a distinct temporal progression in reproductive effort: mating activity was negligible across all groups on Day 1 but increased significantly by Day 3 (Table S1). Under cooler conditions, *Cyp6g1-BA* males demonstrated greater reproductive persistence, maintaining elevated mating frequencies through the final days of the assay (Days 3–5). However, this behavioural advantage was lost under warm conditions, where mating frequency in *Cyp6g1-BA* males declined rapidly after Day 2, leading to the convergence of mating performance between genotypes (Table S1). In contrast, while susceptible *Cyp6g1-M* males were characterized by a consistently low reproductive baseline, their temporal patterns shifted under heat stress. In the warm, insecticide-free condition, *Cyp6g1-M* males exhibited near-total reproductive failure, with almost no mating events initiated across the five-day period. However, under combined heat and insecticide exposure, *Cyp6g1-M* males showed a marginal “stir-up” in activity, with sporadic mating events appearing on Days 2 and 3 that were otherwise absent under heat alone (Table S1).

Collectively, these results indicate that the resistance haplotype affects male mating behaviour in a context-dependent manner. While *Cyp6g1-BA* males show elevated mating activity under cooler thermal conditions, this behavioural advantage is diminished under heat stress. Moreover, the impacts of insecticide exposure are contingent upon temperature, highlighting the layered nature of environmental stress on male reproductive performance (Figure 1, Table 1).

### 3.2. Thermal Stress Reveals Genotype-Specific Differences in Fertilisation Success

**Figure 2:**
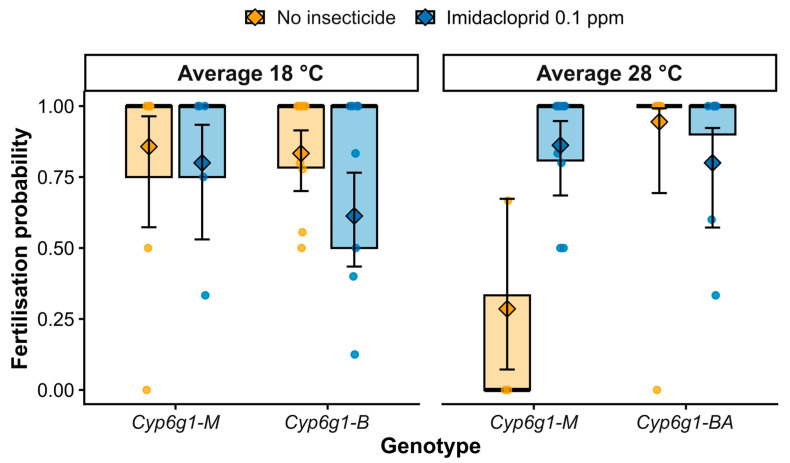
Genotype-specific effects of temperature and insecticide exposure on fertilisation success. Probability that a mating produced progeny for males carrying the susceptible haplotype (*Cyp6g1-M*) or resistance haplotype (*Cyp6g1-BA*) under factorial combinations of temperature (average 18 °C and average 28 °C) and insecticide exposure (no insecticide or imidacloprid 0.1 ppm). Fertilisation success was calculated for each male as the proportion of successful matings across five consecutive daily 4 h mating assays. Points represent individual males (n = 10 males per group), with colours indicating treatment (orange = no insecticide; blue = imidacloprid 0.1 ppm). Boxes show the interquartile range of raw data (without whiskers). Diamonds indicate model-estimated marginal means, and error bars represent 95% confidence intervals derived from a binomial generalized linear model. Predicted probabilities are back-transformed from the logit scale. Full model results are provided in Table 2, with pairwise comparisons and model diagnostics available in Tables S2 and S3, respectively.

Although mating frequency provides an index of behavioural engagement, it does not necessarily reflect functional reproductive output. We therefore next examined fertilisation success, defined here as whether a mating resulted in offspring. Fertilisation success was analysed using a binomial generalized linear model including temperature (T), insecticide exposure (E), genotype (G), and their interactions. The three-way interaction, T × E × G, showed a marginal trend (χ^2^_1_ = 3.56, *p* = 0.059, Table 2). This interaction term was retained in the final model as it represented our *a priori* experimental hypothesis regarding how the *Cyp6g1* genotype influences the response to multiple stressors. Retaining the full factorial design ensures that estimates for main effects and two-way interactions are calculated within the intended experimental framework, providing a more transparent and conservative assessment of the data than a reduced model (Figure 2, Table 2).

Under the cooler conditions, fertilisation success remained high across genotypes in the absence of insecticide (0.83–0.86 probability; Figure 2). Imidacloprid exposure significantly reduced fertilisation success (odds ratio = 3.16, *p* = 0.032, Table S2) in *Cyp6g1-BA* males (0.83 to 0.61; approximately 27% reduction), whereas *Cyp6g1-M* males showed comparatively modest changes (0.86 to 0.80). Thus, under cooler conditions, the resistance haplotype did not enhance fertilisation success and appeared more sensitive to insecticide exposure. In contrast, under warmer conditions, pronounced genotype-specific differences emerged (Figure 2). In the absence of insecticide under warmer conditions, fertilisation probability in *Cyp6g1-M* males declined to 0.29, whereas *Cyp6g1-BA* males maintained high (0.94) fertilisation success (odds ratio = 42.5, *p* = 0.0047, Table S2). This pattern indicates strong thermal sensitivity of reproductive function in susceptible males under no insecticide conditions.

Under combined warm and insecticide exposure, fertilisation probabilities converged across genotypes (0.80–0.86), indicating that the addition of agrochemical stress reduced the genotype-specific divergence observed under heat alone. Together, these findings suggest that while mating behaviour reflects context-dependent energetic trade-offs, fertilisation success exposes a distinct layer of genotype-specific thermal robustness in male reproductive function (Figure 2).

## 4. Discussion

Insect population declines are increasingly attributed to the combined pressures of climate warming and chemical contamination, both of which can impair reproduction before affecting survival [1,2,9]. Here, we show that exposure to elevated temperature during development and early-life reshapes male reproductive performance in a genotype-dependent manner, and that these effects are further modified by sublethal insecticide exposure. Mating frequency varied across genotypes and environmental conditions (Figure 1), reflecting behavioural plasticity in response to developmental stress. In contrast, fertilisation success remained high across most treatment combinations (Figure 2), with a pronounced collapse restricted to susceptible *Cyp6g1-M* males reared under heat in the absence of insecticide. Because reproductive assays were conducted under non-stressful and non-competitive conditions, these differences reflect early-life environmental history effects rather than acute stress during mating. While our experimental design involved exposure to experimental conditions from the embryo stage through the first three days of adulthood, the observed effects likely stem from stress during critical developmental windows, specifically the morphogenesis and initiation of spermatogenesis that begin during early larval stages [32,33]. And while a constant 25 °C control is standard in labs, our 18 °C fluctuating regime served as a robust functional baseline, with fertilization success remaining consistently high (>0.80 probability) without insecticide exposure. This confirms our cooler condition as a non-stressful reference for measuring heat stress while maintaining ecological validity, which constant temperatures fail to capture. Experimental studies have shown that thermal exposure at different developmental stages can impair reproductive capacity later in life, particularly through effects on spermatogenesis and gamete function [11]. Together, our findings demonstrate that layered stress during early-life can generate persistent and genotype-specific consequences for male fertility.

Male fertility is widely recognised as more thermally sensitive than survival in *Drosophila* and other ectotherms [3,10,34,35]. Spermatogenesis and sperm function are particularly vulnerable to elevated temperatures, often at thresholds well below lethal limits [10]. Developmental environments can shape adult physiological architecture and life-history allocation, with persistent consequences for performance under later stress [36]. Notably, when heat was combined with insecticide exposure, the sharp reduction in fertilisation success observed in susceptible *Cyp6g1-M* males under heat alone was no longer evident. While the interaction between genotype and these stressors was only marginally significant (*p* = 0.059) and warrants a cautious interpretation, this qualitative shift resulted in similar fertilisation probabilities across genotypes (Figure 2). This non-additive pattern indicates that agrochemical exposure modified the magnitude of the thermal response rather than simply intensifying stress effects, underscoring the importance of examining multiple stressors simultaneously when assessing reproductive limits.

One alternative explanation for the improved fertility of *Cyp6g1-M* males under warmer conditions and low imidacloprid dose is developmental selection, where high juvenile mortality might leave only the most robust individuals to eclose. However, our assessment of developmental success showed that embryo-to-adult viability remained relatively high across all groups (Table S4). The survival levels suggest that the adults used in our assays were representative of their treatment cohorts and that the results reflect physiological plasticity rather than a selective bottleneck. A potential mechanism for this observed plasticity is hormesis.

Hormetic responses describe processes whereby mild stress induces compensatory physiological adjustments that enhance subsequent performance [37,38]. In insects, hormesis is thought to involve homeostatic overcompensation and activation of stress-response pathways, although the underlying molecular mechanisms remain incompletely resolved. While we did not directly quantify physiological or molecular markers of stress (e.g., antioxidant enzyme activity or heat shock protein expression), our results suggest a hormetic effect. Low-dose imidacloprid exposure has been shown to induce oxidative stress, triggering antioxidant responses and metabolic reprogramming [4]. Similarly, heat stress in insects, including *Drosophila*, disrupts mitochondrial function and promotes the generation of reactive oxygen species [39,40,41]. In *Drosophila*, the *CncC*/*Nrf2* pathway is a central regulator of antioxidant responses and also coordinates the expression of detoxification genes such as *Cyp6g1* [9,42]. The resistant *Cyp6g1-BA* genotype is characterised by elevated constitutive investment in xenobiotic defence, whereas the susceptible *Cyp6g1-M* genotype is more likely to rely on inducible responses. The recovery of fertility observed in *Cyp6g1-M* males under combined warm temperature and insecticide exposure suggests that imidacloprid-induced activation of stress-response pathways may confer cross-protection against heat-induced oxidative damage. Given that spermatogenesis in *Drosophila* is highly sensitive to oxidative stress [43,44], low-dose imidacloprid may prime antioxidant and stress-response pathways, such that subsequent or concurrent heat exposure elicits a more effective defensive response, reducing oxidative damage to developing sperm in *Cyp6g1-M* males. Although this interpretation remains speculative, it provides a biologically plausible framework in which mild activation of detoxification and redox pathways during development recalibrates the physiological state in a manner that enhances reproductive performance later in life.

In contrast to *Cyp6g1-M* males, the resistance-associated *Cyp6g1-BA* haplotype maintained fertilisation success across environmental conditions, indicating differential reproductive robustness between genotypes. While *Cyp6g1* is best characterised for its role in xenobiotic detoxification [17,18], variation at this locus has been linked to broader life-history traits, including fecundity, body size, and developmental timing [23,24,25]. The mechanisms underlying these pleiotropic effects remain unresolved. Our results indicate that resistance-associated genetic variation can alter reproductive sensitivity to environmental stress even in the absence of acute insecticide exposure during adulthood. Adaptive alleles favoured under chemical pressure may therefore reshape physiological stress architecture in ways that become evident only under layered environmental challenges.

Across most treatments, behavioural engagement and fertilisation success were broadly aligned; however, under heat stress in susceptible males reared without insecticide, the proportional reduction in fertilisation success was more severe than the reduction in mating frequency. This suggests that while heat suppresses mating activity, it has an even more profound impact on gamete viability or transfer, indicating that behavioural measures alone may underestimate the total reproductive impairment caused by thermal stress. Because reproduction often constrains population growth more strongly than survival [2,8], genotype-dependent reductions in fertility can directly influence population dynamics and allele frequency trajectories. In natural systems, insects are exposed to multiple concurrent stressors beyond those investigated here, including parasites, nutritional limitation, and habitat loss [1,5]. If fertility limits vary across genotypes and are reshaped by developmental stress interactions, population responses to climate warming and agrochemical exposure will depend not only on average tolerance but also on underlying genetic composition, with potential consequences for long-term demographic stability.

## 5. Conclusions

Our study demonstrates that heat and agrochemical stress experienced during early-life can expose genotype-dependent limits to male fertility. In our experimental design, males were reared under different thermal and insecticide environments from embryo to adulthood but were allowed to mate under benign conditions without heat or insecticide exposure. Consequently, differences in reproductive performance reflect developmental and early-life effects, indicating that environmental history can alter physiological state and reproductive capacity later in adulthood. The interaction between thermal and agrochemical exposure was non-additive and contingent on genetic background, highlighting how adaptive alleles associated with insecticide resistance can modify reproductive responses to environmental stress. Because fertility directly constrains population growth, genotype-specific reductions in reproductive performance have the potential to influence both demographic stability and allele frequency trajectories in natural populations. Integrating developmental stress history, resource allocation dynamics, and genetic variation will therefore be essential for predicting how insect populations respond to interacting pressures of climate warming and environmental contamination.

## Figures and Tables

**Table 1:** Effects of temperature, insecticide exposure, and genotype on male mating frequency. Poisson generalized linear mixed model (GLMM) of daily mating counts. Effects of temperature (average 28 °C vs. average 18 °C), insecticide exposure (imidacloprid 0.1 ppm vs. no insecticide), genotype (resistant *Cyp6g1-BA* haplotype vs. susceptible *Cyp6g1-M* haplotype), and their interactions are shown. To account for variation between mating frequency and detect temporal trends, day (1–5) was included as a fixed effect to model temporal variation in mating frequency. Effect sizes are presented as incidence rate ratios (IRRs) representing multiplicative changes in daily mating frequency relative to the reference category (18 °C, no insecticide, *Cyp6g1-M*). Values > 1 indicate increased mating frequency and values < 1 indicate reduced mating frequency. Confidence intervals represent model-based 95% intervals. Significance of interaction terms and the temporal factor (day) was assessed using likelihood ratio tests (χ^2^). The χ^2^ value for the genotype main effect (5.33) was derived from the Wald statistic (z^2^). Male ID was included as a random effect to account for repeated measures across days.

Term	IRR	95% CI	df	χ^2^	*p*-Value
Temperature (T; 28 °C vs. 18 °C)	0.50	0.20, 1.24	1	2.24	0.134
Insecticide Exposure (E; imidacloprid vs. none)	1.07	0.52, 2.22	1	0.03	0.853
genotype (G; *Cyp6g1-BA* vs. *Cyp6g1-M*)	3.43	1.89, 6.22	1	16.46	<0.001
Day (D)	-	-	4	32.17	<0.001
T × E	3.87	1.29, 11.63	1	5.80	0.016
T × G	0.75	0.26, 2.16	1	0.28	0.594
E × G	0.60	0.26, 1.42	1	1.34	0.247
T × E × G	0.44	0.12, 1.72	1	1.38	0.240

**Table 2:** Effects of temperature, insecticide exposure, and genotype on fertilisation success. Binomial generalized linear model of fertilisation success. Effects of temperature (average 28 °C vs. average 18 °C), insecticide exposure (0.1 ppm imidacloprid vs. no insecticide), genotype (resistant *Cyp6g1-BA* haplotype vs. susceptible *Cyp6g1-M* haplotype), and their interactions are shown. The response variable was modelled as the number of successful matings relative to the total number of matings per male. Effect sizes are presented as odds ratios (ORs) representing multiplicative changes in the odds that mating produced offspring relative to the reference category (18 °C, no insecticide, *Cyp6g1-M*). Values > 1 indicate increased odds of fertilisation success and values < 1 indicate reduced odds. Significance of the three-way interaction was assessed using a likelihood ratio test (χ^2^). Confidence intervals represent model-based 95% intervals. χ^2^ statistics correspond to likelihood ratio tests for the three-way interaction. Lower-order terms are derived from Wald statistics (z^2^).

Term	OR	95% CI	χ^2^	*p*-Value
Temperature (T; 28 °C vs. 18 °C)	0.07	0.01, 0.52	5.71	0.017
Insecticide Exposure (E; imidacloprid vs. none)	0.67	0.08, 4.73	0.16	0.685
Genotype (G; *Cyp6g1-BA* vs. *Cyp6g1-M*)	0.83	0.12, 3.91	0.05	0.831
T × E	23.44	1.59, 452.17	5.00	0.025
T × G	51.00	3.05, 1917.43	6.21	0.013
E × G	0.48	0.05, 5.08	0.43	0.512
T × E × G	0.03	0.0005, 1.14	3.56	0.059

## Data Availability

The original contributions presented in this study are included in the article/Supplementary Materials. Further inquiries can be directed to the corresponding authors.

## Supplementary Materials

The following supporting information can be downloaded at: https://www.mdpi.com/article/10.3390/insects17040426/s1, Table S1. Raw data. Table S2. Pairwise comparisons of male reproductive performance across experimental treatments. Results are derived from post-hoc comparisons (Tukey’s HSD) following Generalized Linear Mixed Models (GLMM) for mating frequency and Generalized Linear Models (GLM) for fertilization success. Table S3. Model diagnostics and goodness-of-fit statistics for reproductive performance analyses. This table summarizes the parameters used to validate the statistical rigor of the models reported in the main text. Table S4. Embryo-to-adult viability.
